# Increased prevalence of human papillomavirus in fresh tissue from penile cancers compared to non-malignant penile samples: a case-control study

**DOI:** 10.1186/s12885-022-10324-w

**Published:** 2022-11-28

**Authors:** Sinja Kristiansen, Carina Bjartling, Christian Torbrand, Diane Grelaud, Martin Lindström, Åke Svensson, Ola Forslund

**Affiliations:** 1 Department of Dermatology and Venereology, Lund University, Skane University Hospital, Jan Waldenströmsgata 16, 214 28 Malmö, Sweden; 2 Department of Obstetrics and Gynecology, Lund University, Skane University Hospital, Malmö, Sweden; 3 Lund University, Department of Urology, Helsingborg Hospital, Helsingborg, Sweden; 4 Lund University, Institution of Translational Medicine, Malmö, Sweden; 5 Department of Pathology, Skane University Hospital and Regional Laboratories, Malmö, Sweden; 6 Lund University, Department of Medical Microbiology, Laboratory Medicine, Lund, Sweden; 7 Clinical Microbiology, Infection Prevention and Control, Office for Medical Services Region Skane, Kristianstad, Sweden

**Keywords:** Penile cancer, Human papillomavirus, HPV. HPV16 expression, Non-malignant penile controls

## Abstract

**Background:**

HPV has been detected in approximately 50% of invasive penile cancers but with a large span between 24 and 89%, most likely due to different types of tumors and various methods for HPV analysis. Most studies of HPV in penile cancer have been performed using paraffin-embedded tissue, argued to be at risk for contaminated HPV analysis. Viral activity of HPV, by the use of HPV mRNA expression is well studied in cervical cancer, but seldom studied in penile cancer.

The aim was to determine prevalence of HPV types in fresh tissue of penile cancers compared to non-malignant age-matched penile controls. Additional aims were to analyze the viral expression and copy numbers of HPV16-positive tumors and 10 mm adjacent to the tumor.

**Methods:**

Fresh tissue from penile cancer cases was biopsied inside the tumor and 10 mm outside the tumor. Controls were males circumcised for non-malignant reasons, biopsied at surgery.

PCR and Luminex assays were used for identification of HPV types. HPV16-positive samples were investigated for copy numbers and expression of HPV16-mRNA.

**Results:**

Among tumors (*n =* 135) and age-matched controls (*n =* 105), HPV was detected in 38.5% (52/135) and 11.4% (12/105), respectively (*p* < 0.001), adjusted odds ratio 12.8 (95% confidence interval 4.9–33.6). High-risk HPV types were found in 35.6% (48/135) of tumors and 4.8% (5/105) of controls (*p* < 0.001). Among tumors and controls, HPV16 was present in 27.4% (37/135) and 1% (1/105), respectively (p < 0.001). Among HPV16-positive penile cancers, mean HPV16 viral copy/cell was 74.4 (range 0.00003–725.4) in the tumor and 1.6 (range 0.001–14.4) 10 mm adjacent from the tumor. HPV16-mRNA analysis of the tumors and 10 mm adjacent from the tumors demonstrated viral activity in 86.5% (32/37) and 21.7% (5/23), respectively.

**Conclusions:**

The prevalence of HPV was significantly higher in penile cancer (38.5%) than among age-matched non-malignant penile samples (11.4%). HPV16 predominates (27.4%) in penile tumors. HPV16 expression was more common in penile cancer than in adjacent healthy tissue, strongly suggesting an etiological role for HPV16 in the development of penile cancer.

**Supplementary Information:**

The online version contains supplementary material available at 10.1186/s12885-022-10324-w.

## Background

Penile cancer is a rare cancer worldwide, with an incidence of 0.45–1.7/100000 in Europe [1]. In Sweden the overall five-year relative survival is 82% [2]. Histologically, penile cancer is classified as squamous cell carcinoma (SCC) in about 95% of cases [3]. Different histological morphologies of SCCs are observed in penile cancer with usual type (keratinized), verrucous, basaloid, and warty being most prevalent. Human papillomavirus (HPV) is frequently detected in basaloid, warty and warty-basaloid penile SCC-lesions [4].

Two major pathways of malignant transformation to penile cancer have been described; one HPV-driven and the other derived from inflammatory skin diseases such as lichen sclerosus (LS) and lichen planus (LP) [5]. Other risk factors are phimosis, balanitis, smoking, treatment with Psoralen plus ultraviolet light A (UVA) and immunosuppression following organ transplantation [6–9].

HPV has been detected in approximately 50% of invasive penile cancers but with a large span between 24 and 89%, most likely due to different types of tumors and various methods for HPV analysis [10–13]. HPV types are classified as low-risk (LR) or high-risk (HR) depending on their oncogenic potential [14]. According to WHO, the following HR types are oncogenic HPV16, 18, 31, 33, 35, 39, 45, 51, 52, 56, 58, and 59 [14]. Most studies of HPV in penile cancer have been performed using paraffin-embedded tissue, argued to be at risk for contaminated HPV analysis [15, 16]. Only a few studies have been performed using fresh tissue from penile cancer [12, 17, 18].

Viral activity of HPV, by the use of HPV mRNA expression is well studied in cervical cancer [19, 20], but seldom studied in penile cancer [21].

To the best of our knowledge no study has been published analyzing HPV among biopsied fresh tissue from both penile cancer and from age-matched penile non-malignant samples.

The primary aim of this study was to determine the prevalence of different HPV types in fresh tissue of invasive penile cancer and 10 mm adjacent to the tumor, and to compare that to corresponding HPV prevalence in fresh biopsies from age-matched non-malignant penile controls. A secondary aim was to conduct a blinded review of subtype of penile cancer and correlate penile cancer subtype to the prevalence of HPV. Furthermore, we aimed to determine copy numbers, status of integration and viral activity among HPV16-positive samples inside the tumor and 10 mm adjacent to the tumor.

## Materials and methods

The study was approved by the ethical board in Lund with diary number 2015/907. Inclusion criteria were men over 18 years, having penile cancer surgery at Skane University Hospital (SUH) in Sweden between 22nd of June 2015 and 10th of August 2021. SUH is one of two national referral centers for penile cancer. Excised tumor tissues were transported fresh to the Department of Pathology, SUH and a 3 mm single-use punch biopsy was taken from the tumor and submerged in 1 mL RNAlater (Ambion). Another 3 mm single-use punch biopsy was taken 10 mm outside the macroscopic margins of the tumor and submerged in 1 mL RNAlater (Ambion).

Controls comprised age-matched men over 18 years, circumcised for non-malignant reasons at urological departments in Skane. At surgery, a biopsy of approximately 5 mm was taken from excised penile skin, and submerged in 1 mL RNAlater (Ambion) as described [22].

The biopsies were transported to the Department of Microbiology where they were transferred to 1 mL GITS-solution (4 M guanidinium thiocyanate, 22 mM NaCitrate and 5% Sarcosyl (N-Lauroylsarcosine sodium salt) and 1% mercaptoethanol) and incubated at room temperature overnight. Then DNA was extracted with the Total NA-kit (Roche, Stockholm, Sweden) using MagNA Pure LC (200 μL input and 100 μL output). Sample adequacy was assessed by testing 5 μL of the sample for the human beta globin gene with a real-time PCR [23]. Simultaneous identification of 40 genital HPV types was carried out by MGP-PCR in 25 μL, containing 5 μL of extracted material and subsequent Luminex analysis [24, 25], including probes for 40 HPV types: 6, 11, 16, 18, 26, 30, 31, 33, 35, 39, 40, 42, 43, 45, 51, 52, 53, 54, 56, 58, 59, 61, 62, 66, 67, 68 (a and b), 69, 70, 73, 74, 81, 82, 83, 85, 86, 87, 89, 90, 91 and 114.

The integration of HPV-DNA into the human genome most frequently disrupts the E2 gene [26]. As a surrogate marker for the physical status of HPV16, the quantity of HPV16 E2 gene was determined as described by Letsolo et al. [27]. We used the mean log10 values of E2 and E7 copy numbers from each sample and calculated the ratio of E2/E7 gene copy numbers of HPV16 to investigate the presence of integrated, mixed and episomal forms of HPV16. HPV16 was classified as follows: ‘integrated’ when no E2 copy numbers could be detected and E7 copy numbers were present; ‘mixed status’ when E2/E7 ratios were 0.1–0.8; and ‘episomal status’ when the presence of E2/E7 copy number ratio was > 0.8. The samples were analyzed in duplicate. In addition, another aliquot (200 μL) of the GITS-lysate was used for mRNA extraction using the Oligotex Direct mRNA Mini Kit (Qiagen). The extraction was performed according to the manufacturer’s protocol for isolation of PolyA mRNA from animal tissues. The mRNA was eluted by adding 45 μL of Oligotex elution buffer (70 °C) to the column and centrifuging for 1 minute at maximum speed. Purified mRNA was stored at − 80 °C until use. Quantitative PCR of HPV16 E7 mRNA was analyzed in triplicate and performed as previously described [27]. To compensate for the smaller elution volume of the mRNA extraction (45 μL) compared to that of the DNA extraction (100 μL), the HPV16 mRNA copy numbers were divided by 2.5. The HPV16 mRNA expression level was given as HPV16 mRNA copy numbers per HPV16-DNA copy.

Histopathological diagnosis and classification of the subtype of penile cancer was retrospectively reviewed by two experienced pathologists subspecialized in uropathology and participating in the national multidisciplinary team conference of penile cancer in Sweden. A recent study showed that pathologists who have experience of penile cancer diagnostics have a good concordance in identifying HPV-related and non-HPV-related histological subtypes of penile cancer [28].

The glass slides were retrieved from the pathology archive, and one representative slide of the tumor was chosen for each case in hematoxylin-eosin (H&E) stain. Assessment of histological subtype and histological grade was carried out using glass slides (106 cases) and high-resolution digital slides (40 cases). The slides were scanned using NanoZoomer S360 (S60 for large histologic sections) by Hamamatsu, and Sectra IDS7 software. The pathologists were blinded to the results of the HPV detection by PCR in the tumor material to avoid any bias in tumor assessment. The histological subtypes were determined according to the WHO criteria in “Classification of tumors of the urinary system” and the International Society of Urological Pathology recommendations (2016) [4, 29]. For classification of stage TNM 8 was used. Following the separate assessment of all cases, the results were compared to identify eventual discrepancies, which were found in six cases where the diagnosis was difficult to make without HPV status. p16INK status was used in five cases and HPV result in one case and thereby the pathologists agreed upon the final diagnosis.

Circumcised skin was subjected to routine histopathological examination at the Department of Pathology at SUH. All specimens were stained with haematoxylin and eosin. All diagnoses were performed by the expert team of six uro-pathologists belonging to the Swedish national Penile Cancer Center in Malmö.

All cases and controls completed a questionnaire, regarding medical history, medication, smoking habits, number of sexual partners and former symptoms and procedures performed on the penis (Supplementary 1). Controls completed the questionnaire in connection with the circumcision, while penile cancer cases completed the questionnaire after surgery. If the questionnaire was not returned, a new letter was sent to the patient and if not returned, the patient was informed by phone and if necessary, a further questionnaire was sent.

### Statistical analysis

The statistical analysis was performed using SPSS, version 26, IBM Statistics, IBM Corp., Armonk, NY, USA. Correlation was calculated with Chi-square tests and Fisher’s exact test in small numbers. When *p* < 0.2, odds ratios were calculated with multiple logistic regression adjusted for > 10 sexual lifetime partners, smoking, former smoking, former skin disease, former phimosis, former penile biopsy, former penile surgery and former genital warts.

## Results

**Fig. 1:**
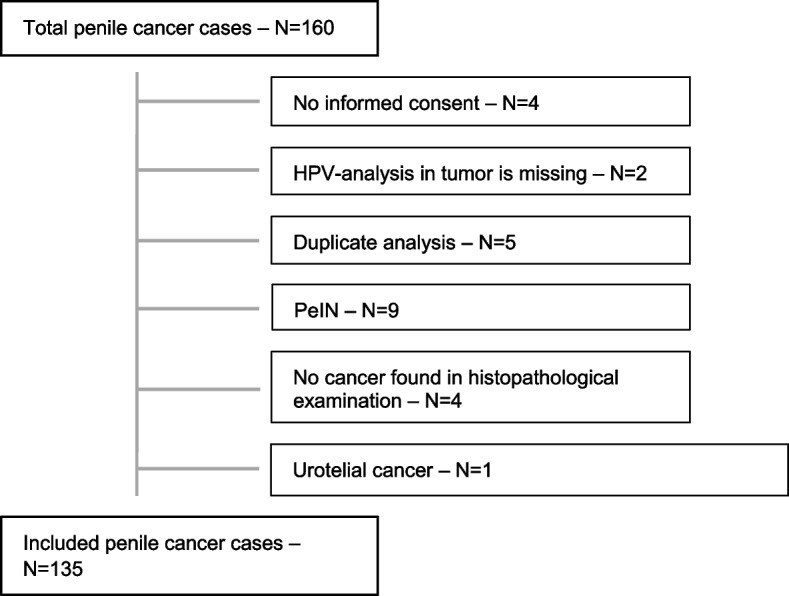
Included and excluded penile cancer cases

The study included 135 men with penile cancer, with a median age of 72 years (interquartile range 66–79 years) (Fig. 1). Histopathological tumor stage according to the TNM-classification was pT1 in 25.2% (34/135) of cases, pT2 in 48.1% (65/135), pT3 in 25.9% (35/135) and pT4 in 0.7% (1/135). The 135 penile cancer cases, were age-matched within +/− 2 years with 105 controls circumcised for non-malignant reasons, median age 71 years (interquartile range 62–76 years) (Table 1). The vast majority of both cases and controls were heterosexual, having sex only with women. A history of phimosis was more common among the penile cancer cases than among the controls, 55.1 and 37.6%, respectively, (*p =* 0.017). Having previously had a punch biopsy taken for histopathologic evaluation was more common among the penile cancer cases (20.5%) than the controls (2%), (*p* < 0.001). Previous surgery on the penis was more common among the penile cancer cases (31.5%) than among the controls (11.9%), (*p =* 0.001). Having had a diagnosis of penile cancer before was only seen in the penile cancer cases (11.6%) and not among the controls 0%.

**Table 1:** Mean age among penile cancer cases and controls and patient data from medical questionnaire. Statistical calculations with Chi-square tests and binary logistic regression with crude OR

	Penile cancer cases (*n =* 135)		Controls (*n =* 105)		***P***-value (Chi-square tests)	OR (95% CI)
**Mean age years (median)**	70.9 (72)		68 (71)			
**Taking medication**	No.	(%)	No.	(%)		
Any medication	75	55.6	69	65.7	0.9	1.0 (0.5–1.7)
No medication	34	25.2	30	28.5		
Missing	26	19.3	6	5.7		
**Smoking**						
Present smoker	15	11.1	6	5.7	0.06	2.5 (0.9–6.7)
Former smoker	69	51.1	49	46.7	0.06	1.7 (1.0–3.1)
Non-smoker	95	70.4	95	90.5		
Missing	25	18.5	4	3.8		
**Lifetime sexual partners**						
0–5	60	44.4	59	56.2	0.6	0.9 (0.5–1.5)
6–10	18	13.3	17	16.2	0.9	1.0 (0.5–2.0)
11–15	10	7.4	11	10.5	0.7	0.8 (0.3–2.1)
> 15	17	12.6	10	9.5	0.2	1.7 (0.7–3.9)
Missing	30	22.2	8	7.6		
**Sexual orientation**						
Heterosexual	106	78.5	98	93.3	0.6^a^	2.2 (0.2–24.2)
Homosexual	0	0	2	1.9	0.2^a^	
Bisexual	1	0.7	0	0		
Sex with transgender persons	0	0	0	0		
Missing	28	20.7	5	4.8		
**History of this condition on penis** (patients can fill in more than one disease/symptom/procedure)						
Phimosis	60	44.4	38	36.2	0.017	2.0 (1.1–3.4)
Itch	28	20.7	19	18.1	0.2	1.5 (0.8–2.8)
Skin disease	8	5.9	9	8.6	0.6	0.8 (0.3–2.1)
Genital warts	3	2.2	3	2.9	0.9	0.9 (0.2–4.6)
Penile cancer	13	9.6	0	0	< 0.001	
Pathologic histologic examination	23	17.0	2	1.9	< 0.001	12.8 (2.9–55.8)
Former surgery	35	25.9	12	11.4	0.001	3.4 (1.7–7.0)

^a^ Fisher exact test

*OR *Odds ratio, *CI *Confidence interval

### HPV

HPV was detected in 38.5% (52/135) of penile cancers and in 11.4% (12/105) of the controls (*p* < 0.001) (Table 2). HR HPV in the tumor was seen in 35.6% (48/135) and in 4.8% (5/105) of the controls (p < 0.001). HPV16 was found in the tumor in 27.4% (37/135) and 1% (1/105) of the controls, respectively (p < 0.001). The frequencies of HPV types are shown in Table 3. Crude OR for HPV among penile cancer tissue compared to penile non-malignant controls was 4.9 (95% CI 2.4–9.7). The adjusted OR was 12.8 (95% CI 4.9–33.6).

**Table 2:** HPV found in penile cancer cases and controls, *p*-values calculated with Chi-squared tests and binary logistic regression with crude odds ratios and 95% confidence interval

	All penile cancer cases ***N =*** 135 (%)	All cases, 10 mm adjacent to tumor ***N =*** 132 (%)	Age-matched controls ***N =*** 105 (%)	***P***-value (Chi-2)	Crude OR ^**b**^ (95% CI)
**Any HPV type**	38.5 (52/135)	30.3 (40/132)	11.4 (12/105)	< 0.001	4.9 (2.4–9.7)
**High risk HPV type**	35.6 (48/135)	25.8 (34/132)	4.8 (5/105)	< 0.001^a^	16.8 (3.6–78.0)
**Low risk HPV only**	3.0 (4/135)	4.5 (6/132)	6.7 (7/105)	< 0.001^a^	0.06 (0.01–0.3)
**HPV16**	27.4 (37/135)	17.4 (23/132)	1.0 (1/105)	< 0.001^a^	27.1 (3.2–229.1)
**Multiple HPV types**	3.7 (5/135)	5.3 (7/132)	1.0 (1/105)	1.0^a^	1.2 (0.1–11.0)

^a^ Fisher exact test

^**b**^ Crude odds ratios after binary logistic regression with all penile cancer cases and age-matched controls

**Table 3:**
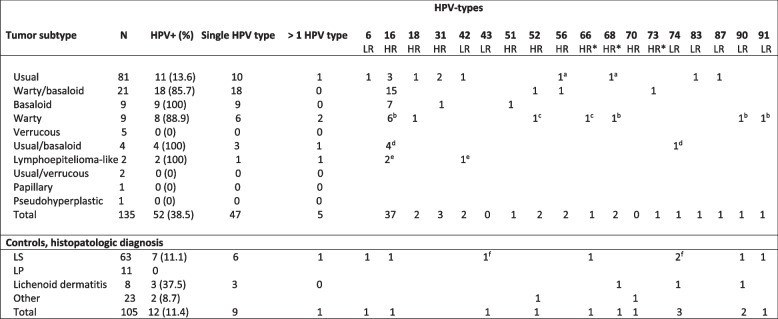
HPV type distribution among 135 penile cancer subtypes and in 105 non-malignant penile controls

*HR *High risk, *LR *Low risk. *HPV66, 68 and 73 were here classified as high risk, according to WHO they are potential high risk types [14]

^a, c, d, e, f^ Samples double HPV positive for marked HPV types. ^b^One sample was positive for 4 HPV types

Adjacent to the tumor, HPV was found in 30.3% (40/132) of the samples. HR HPV types were detected in 25.8% (34/132) of the samples, with HPV16 present in 17.4% (23/132). Only LR HPV types were seen in 4.5% (6/132) of the samples. All cases of HPV occurrence adjacent to the tumor also had at least one HPV type identical to that in the tumor, except for one case where HPV6 was adjacent to the tumor but HPV-negative in the tumor. In two samples 10 mm adjacent to the tumor, the HPV analysis was not adequate, due to no detectable human DNA in the sample. In one sample 10 mm adjacent to the tumor, the analysis was not performed.

In all HPV16-positive cases, the E2/E7 ratio showed HPV16 to be episomal in 59.5% (22/37) of the tumors and in 69.6% (16/23) adjacent to the tumors. HPV was integrated in 27% (10/37) and 13% (3/23) in the tumor and adjacent to the tumor, respectively. Mixed forms of HPV16 were seen in 13.5% (5/37) in the tumor and in 4.3% (1/23) adjacent to the tumor. A weak E7 signal only was seen in one case 10 mm adjacent to the tumor. Viral activity shown by HPV16 mRNA expression was detected in 86.5% (32/37) of the tumors, with a mean mRNA expression of 15.1 (median 8.9) HPV16 mRNA copies/HPV16 DNA copy. Adjacent to the tumor, 21.7% (5/23) of the samples showed viral activity, with a mean mRNA expression of 73.1 (median 7.4) HPV16 mRNA copies/HPV16 DNA copy. In the HPV16-positive tumors the mean viral copy/cell was 74.4 (median 6.5, range 0.00003–725.4 viral copies/cell) and in the HPV16-positive adjacent tissue it was 1.6 (median 0.1, range between 0.001 and 14.4 viral copies/cell) (Supplementary 2).

### Histopathological subtype of penile cancer and diagnosis in non-malignant controls

**Fig. 2:**
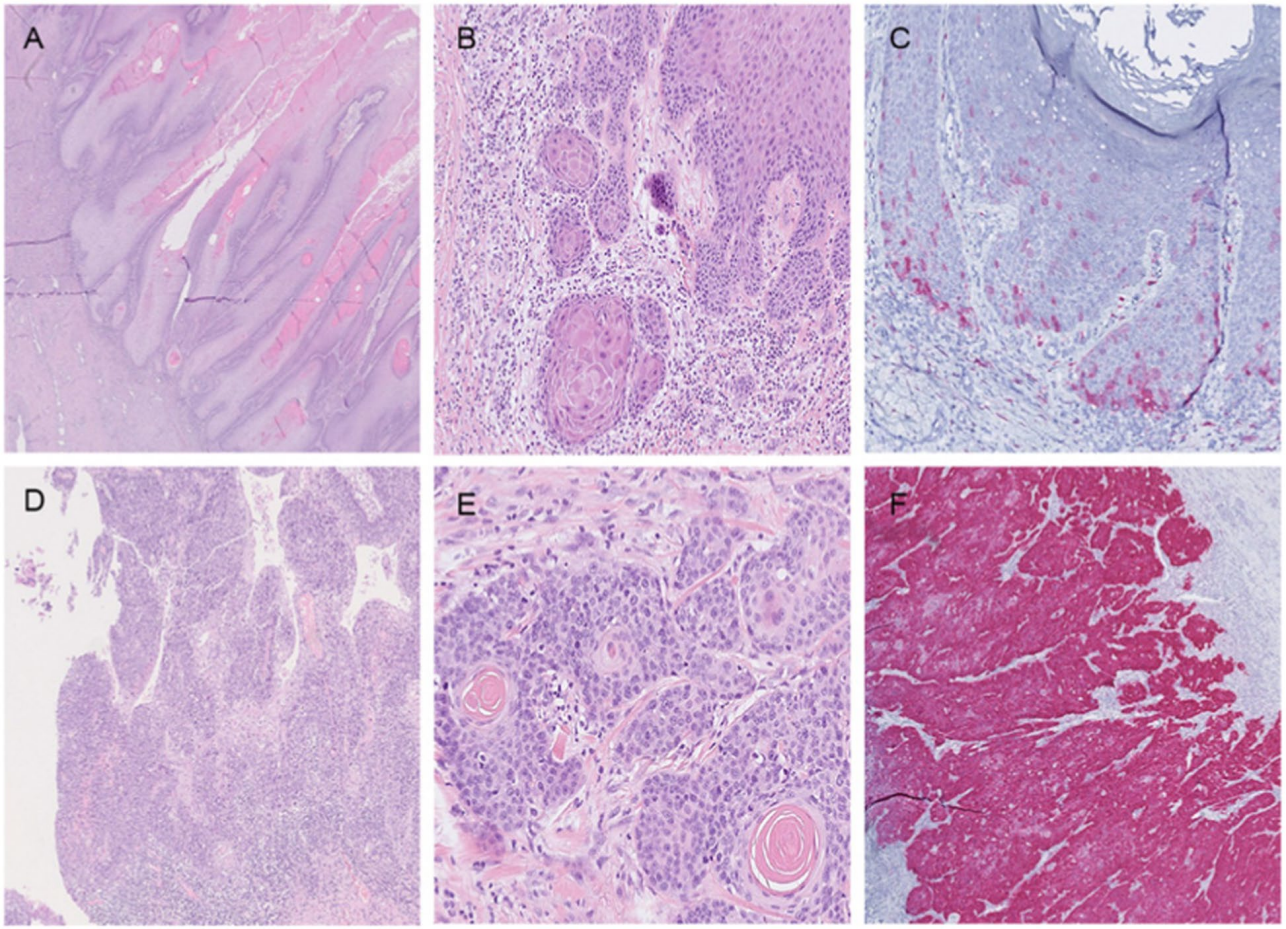
**A** (top left). Verrucous carcinoma. Note exophytic growth pattern and pushing borders. **B** (top middle). Invasive squamous cell carcinoma, usual type. Note basal and parabasal atypia with retained keratinization and a mild stromal response. **C** (top right). P16-immunohistochemical staining did in rare instances, showed a non-specific focal and cytoplasmatic positivity in cancer of usual types and associated differentiated PeIN. **D** (bottom left). Warty carcinoma. Exophytic growth pattern with complex fused papillae and poorly defined borders. **E** (bottom middle). Invasive basaloid carcinoma. Note abrupt keratinization and slight stromal response. **F** (bottom right). Typical positive reaction for P16 in basaloid carcinoma

Classification of subtypes of penile cancer showed that the usual type was seen in 60% (81/135) of cases (Table 3). The second most prevalent subtype was warty-basaloid in 15.6% (21/135) of cases, followed by basaloid and warty, both seen in 6.7% (9/135) each. The verrucous type was seen in 3.7% (5/135) of cases and other types were seen in less than 3%. For histopathologic slides of different subtypes see Fig. 2. HPV was most frequent in basaloid subtypes of penile cancer compared to other subtypes, seen in 100% (9/9) and 34.1% (43/126), respectively (*p* < 0.001). The subtype of penile cancer where HPV was second most common was in warty subtypes, where HPV was seen in 88.9% (8/9) compared to in 34.9% (44/126) of other subtypes (*p =* 0.002). Warty-basaloid subtypes of penile cancer were HPV-positive in 85.7% (18/21) compared to 29.8% (34/114) in other subtypes (*p* < 0.001) with an increased adjusted OR for HPV of 9.0 (95% CI 1.4–56.2, *p =* 0.019). In the usual subtype of penile cancer, HPV was found in 13.6% (11/81) (p < 0.001). Decreased risk for HPV was seen in the usual subtype of penile cancer with an adjusted OR of 0.03 (95% CI 0.007–0.15, *p* < 0.001).

The circumcised tissue from the non-malignant controls was sent for histopathological routine evaluation showing LS in 60% (63/105) and LP in 10.5% (11/105). Lichenoid dermatitis was seen in 7.6% (8/105). In LS, HPV was seen in 11.1% (7/63) and in 37.5% (3/8) of lichenoid dermatitis, but HPV was not found in LP (Table 3). Phimosis was the most common indication for circumcision (84.8%) (Supplementary 3).

## Discussion

To the best of our knowledge, this is the largest study using fresh penile cancer tissue for HPV analysis, and the only one with age-matched penile controls. We observed that HPV was three times more common in invasive penile cancer (38.5%) than in non-malignant controls (11.4%), and that HR HPV was seven times more common in invasive penile cancer (35.6%) than in non-malignant controls (4.8%). HPV16 was the predominant HPV type.

Only a few other studies have searched for the presence of HPV in fresh penile tissue. Martins et al. found HPV in 89% (49/55) of penile cancer cases, which was substantially higher than our HPV prevalence (38.5%) among invasive penile cancer cases [12]. In comparison to our PCR method, they used a different approach with nested PCR, which in general has high analytical sensitivity but could be affected by an increased risk of cross-contamination [30]. However, our result is more like that of Levi et al., who found 56% HPV in fresh penile cancer tissue in Brazil [17]. In addition, our HPV prevalence (38.5%) among the penile cancer cases is in alignment with the average HPV prevalence of around 50% among penile cancers [13] as well as that of cancer of the vulva, which is in the range between 34 and 45% [31].

In our study, HPV16 was found to be the predominant HPV type, in agreement with other studies of HPV in penile cancer [13].

In the present study we analyzed HPV also outside of the tumor in order find out possible differences between tumors and adjacent healthy tissue. Interestingly, HPV was relatively frequently detected (30.3%) in the tissue adjacent to the tumors. However, there was a tendency of a higher proportion of HPV16 positive tumors compared to that of the adjacent tissue (27.4% vs. 17.4%, *p =* 0.0573). Furthermore, our finding of a higher prevalence of HPV16 viral activity in penile cancer cases (86.5%), than in samples taken adjacent to the tumor (21.7%), suggests that HPV is more active in the tumor and probably has a role in the malignant transformation process.

The observed mean HPV16 viral load (74.4 virus copies per cell) in the tumor was similar to that of Heideman et al., who found a mean HPV16 load of 72 copies per cell [21]. Other studies on viral load in penile cancer are scarce, but viral load in penile cancer appears to be low compared to viral load in the vulva, where a median of 14,676 HPV16 copies/cell (range between 499 and 1,477,442) was observed [32]. Integration of HPV in our study was 40.5% (15/37), which was much lower than in a study by Huang et al. using high-throughput viral integration detection (HIVID) in frozen specimens from penile cancer surgery and showing integration of HPV in 92.1% (35/38) [33]. Interestingly, and in comparison with cervical cancer, they showed that HPV E2 was significantly less likely to be involved in HPV integration than expected (*p* = 1.25 × 10^− 5^). Next-generation sequencing appears to yield a higher HPV integration rate than the E2/E7 ratio method used in our study, which is affected by limitations such as decreased detection of HPV integration when a large excess (at least 10-fold) of episomal HPV16 is simultaneously present [15].

In our study, the HPV prevalence was highest in basaloid subtypes of penile cancer (100%), followed by warty subtypes (88.9%) and warty-basaloid subtypes (85.7%). This is in alignment, but slightly higher than former studies where a meta-analysis showed the pooled prevalence of HPV to be 84% in basaloid penile cancers, 58.7% in warty subtypes and 75.7% in warty-basaloid subtypes [13].

The strengths of our study were that HPV was analyzed in fresh biopsies both in and adjacent to the tumor, and that biopsied age-matched controls were used. Another strength was that not only the presence of HPV16 DNA was studied; also the viral activity was investigated in HPV16-positive samples, both in and adjacent to the tumor. A further strength is that histological subtypes of penile tumors were reviewed by two experienced uropathologists, blinded to each other.

One limitation was that compared to the controls, who completed the questionnaire at the hospital before the treatment, there was a relatively high proportion of missing questionnaires (Table 1) from the penile cancer patients because the form had to be sent to them after the treatment and was not always returned. Regarding former smokers, another limitation of the questionnaire was that smoked pack-years were not asked for. Further limitations were that a large number of the non-malignant controls did not have normal histological tissue. In 78.1% of the controls, the histological diagnosis was LS, LP or lichenoid dermatitis, which are potential premalignant inflammatory skin diseases, with increased risk of penile intraepithelial neoplasia and penile cancer [34–36]. One further limitation could be the possible discrepancy in location of HPV between cases and controls since the tissue used for HPV analysis in the controls was circumcised foreskin, but the penile cancer tumors had different penile locations.

Knowledge of HPV as a strong risk factor (adjusted OR 12.8) for invasive penile cancer is important in the work of preventing penile cancer. The nine-valent HPV vaccine targets the HPV types HPV6, 11, 16, 18, 31, 33, 45, 52 and 58, covering the majority of HR HPV types found here, which leads to the conclusion that vaccination of boys is important for preventing development of penile cancer. Knowledge about viral activity in penile cancer tumors, further indicates the important role of HR HPV in the malignant transformation.

## Supplementary Information

**Additional file 1.**

**Additional file 2.**

**Additional file 3.**

## Data Availability

The datasets generated and/or analysed during the current study are not publicly available due confidential information about patients but are available from the corresponding author on reasonable request.

## Abbreviations

CI: Confidence interval
HPV: Human papillomavirus
HR: High-risk
LP: Lichen planus
LS: Lichen sclerosus
LR: Low-risk
OR: Odds ratio
SCC: Squamous cell carcinoma
