# Supplementary material for: Increased prevalence of human papillomavirus in fresh tissue from penile cancers compared to non-malignant penile samples: a case-control study
Source: BMC Cancer. 2022 Nov 28;22:1227. doi: 10.1186/s12885-022-10324-w (PMC9703753; doi:10.1186/s12885-022-10324-w)
Supplement: Supplementary file 1 — Additional file 1. [file 12885_2022_10324_MOESM1_ESM.pdf]

Supplementary 1.

The questionnaires were distributed in Swedish. To be supplemented to the article it was translated to English and then back translation to Swedish was performed by a native speaking translator and compared to the Swedish original.

Questionnaire Human papillomavirus and penile cancer

Date	Name	National identification number
....	....	………………………………

Do you take any medication right now? If yes, which one/s? ………………………………………………………...
……

Do you smoke?	Yes ❑	No ❑		
If yes, how many cigarettes per day?	< 5 ❑	5-10 ❑	10-20 ❑	> 20 ❑
If no, are you a former smoker?	Yes ❑	No ❑		
Number of sexual partners over life?	0-5 ❑	6-10 ❑	10-15 ❑	> 15 ❑
With whom do you have sex?	Women ❑	Men❑	Both women and men❑	
Transgender persons❑				

Have you had narrow foreskin before?	Yes ❑	No ❑
Have you had itch on penis?	Yes ❑	No ❑
Have you had a skin disease on penis before?	Yes ❑	No ❑
Have you had a biopsy taken from your penis before?	Yes ❑	No ❑
Have you had cancer on your penis before?	Yes ❑	No ❑
Have you had surgery on your penis before?	Yes ❑	No ❑
Have you had genital warts on your penis before?	Yes ❑	No ❑
