# Supplementary material for: Increased prevalence of human papillomavirus in fresh tissue from penile cancers compared to non-malignant penile samples: a case-control study
Source: BMC Cancer. 2022 Nov 28;22:1227. doi: 10.1186/s12885-022-10324-w (PMC9703753; doi:10.1186/s12885-022-10324-w)
Supplement: Supplementary file 2 — Additional file 2. [file 12885_2022_10324_MOESM2_ESM.pdf]

Supplementary 2.

Histological subtype and HPV16 characteristics of penile cancer.

Histologic subtype	Total HPV 16+	Episomal	Integrated	Mixed	Episomal %	Integrated %	Mixed %	Mean HPV16 copy per cell	Range HPV16 copy per cell	Median HPV16 copy per cell	Mean HPV16 mRNA per HPV16 copy	Range HPV16 mRNA per HPV16 copy	Median HPV16 mRNA per HPV16 copy	Total HPV16 mRNA positivity
**Usual**	3	1	1	1	33	33	33	20.4	1.6 - 43.5	16.2	6.0	0.9 - 14.0	2.9	3
**Basaloid-warty**	15	8	5	2	53	33	13	109.0	0.00003 - 725.4	6.5	22.0	2.5 - 173.2	9.5	13
**Basaloid**	7	5	1	1	71	14	14	54.0	0.7 - 253.3	8.2	7.0	0.6 - 12.8	5.6	5
**Warty**	6	3	2	1	50	33	17	36.0	0.7 - 190.1	2.4	9.8	1.1 - 26.6	8.8	5
**Verrucous (no HPV positivity)**														
**Basaloid/usual**	4	4	0	0	100	0	0	112.5	1.4 - 411.4	18.7	19.6	4.7 - 32.9	20.5	4
**Lymphoepitelioma-like**	2	1	1	0	50	50	0	5.9	3.9 - 7.9	5.9	7.9	5.0 - 10.9	7.9	2
**TOTAL**	**37**	**22**	**10**	**5**	**59**	**27**	**14**	**74.4**	**0.0003 - 725.4**	**6.5**	**15.1**	**0.6 - 173.2**	**8.9**	**32**
