# Supplementary material for: Increased prevalence of human papillomavirus in fresh tissue from penile cancers compared to non-malignant penile samples: a case-control study
Source: BMC Cancer. 2022 Nov 28;22:1227. doi: 10.1186/s12885-022-10324-w (PMC9703753; doi:10.1186/s12885-022-10324-w)
Supplement: Supplementary file 3 — Additional file 3. [file 12885_2022_10324_MOESM3_ESM.pdf]

Supplementary 3.

Clinical diagnoses before circumcision in 105 controls.

**Clinical diagnosis before circumcision**	**N**	**%**
Phimosis	89	84.8
Visible lichenoid skin changes	11	10.5
Recurrent balanitis	2	1.9
Skin thickening after previous circumcision	2	1.9
Short frenulum	1	0.9
**Total**	105	100
